# Primary squamous cell carcinoma of the pancreas with a large pseudocyst of the pancreas as the first manifestation: a rare case report and literature review

**DOI:** 10.1186/s12876-021-01804-7

**Published:** 2021-05-08

**Authors:** Xia Qiu, Yajie Meng, Meiqin Lu, Chuan Tian, Min Wang, Junwen Zhang

**Affiliations:** 1 Department of Gastroenterology, The People’s Hospital of Nanchuan, Nanchuan District, No. 16 South Street, Chongqing, 408400 China; 2 Department of Gastroenterology, The First Affiliated Hospital of Chongqing Medical University, Chongqing, 400016 China

**Keywords:** Squamous cell carcinoma, Pancreas, Pseudocyst, Endoscopic ultrasound-guided fine-needle aspiration

## Abstract

**Background:**

Primary squamous cell carcinoma (SCC) of the pancreas with pseudocysts, especially diagnosed by endoscopic ultrasound-guided fine-needle aspiration (EUS-FNA), is extremely rare.

**Case presentation:**

A 64-year-old man was admitted to our department for abdominal distension. Two months ago, he experienced abdominal pain for 1 day and was diagnosed with acute pancreatitis in another hospital. After admission, laboratory tests showed the following: amylase 400 U/L, lipase 403 U/L, and carbohydrate antigen 19–9 (CA19-9) 347 U/mL. Abdominal computed tomography (CT) revealed pancreatitis with a pseudocyst with a diameter measuring 7 cm. During linear EUS, a large pseudocyst (5.4 × 5.2 cm) was observed in the pancreatic body. EUS-FNA was performed. We obtained specimens for histopathology and placed a plastic stent through the pancreas and stomach to drain the pseudocyst. Puncture fluid examination revealed the following: CA19-9 > 12,000 U/mL carcinoembryonic antigen (CEA) 7097.42 ng/ml, amylase 27,145.3 U/L, and lipase > 6000 U/L. Cytopathology revealed an abnormal cell mass, and cancer was suspected. Furthermore, with the result of immunohistochemistry on cell mass (CK ( +), P40 ( +), p63 ( +), CK7 (−) and Ki-67 (30%)), the patient was examined as squamous cell carcinoma (SCC). However, the patient refused surgery, radiotherapy and chemotherapy. After drainage, the cyst shrank, but the patient died 3 months after diagnosis due to liver metastasis and multiple organ failure.

**Conclusion:**

For patients with primary pancreatic pseudocysts with elevated serum CEA and CA19-9 levels, we should not rule out pancreatic cancer, which may also be a manifestation of primary pancreatic SCC. EUS-FNA is helpful for obtaining histopathology and cytology and thus improving diagnostic accuracy.

## Background

Pancreatic squamous cell carcinoma (SCC) is a primary rare malignancy, accounting for 0.5–2.0% of all malignant pancreatic tumors, and is considered an aggressive subtype with a poor prognosis [1, 2]. In recent years, an increasing number of pancreatic lesions have been diagnosed by endoscopic ultrasound-guided fine-needle aspiration or biopsy (EUS-FNA or EUS-FNB) [3–7]. However, nearly all of the cases were solid or solid-cystic lesions of the pancreas. To our knowledge, there have been no cases of primary SCC of the pancreas with pseudocysts, especially those diagnosed by EUS-FNA. Here, we report a rare case of SCC of the pancreas with a pseudocyst as the main presentation.

## Case presentation

A 64-year-old man was admitted to our department for abdominal distension. Two months ago, he experienced abdominal pain for 1 day and was diagnosed with acute pancreatitis in another hospital. There was no cystic lesion of pancreas at that time. The patient did not present with cutaneous or sclera icterus, fever, melena, swallowing difficulties or diarrhea but did present with weight loss of 2 kg. After admission, the physical examination showed that the patient had tenderness in the upper abdomen, and a mass approximately 5.0 × 6.0 cm in size and soft in texture could be felt in the upper abdomen. The results of other physical examinations were unremarkable. He denied alcohol use or any history of chronic or significant medical or family illnesses. After admission, laboratory tests showed the following: amylase 400 U/L (normal, 0–40 U/L), lipase 403 U/L (normal, 0–60 U/L), carbohydrate antigen 19–9 (CA19-9) 347 U/mL (normal, < 39 U/mL), and albumin 31.4 g/L. Other routine laboratory tests did not indicate any abnormalities.

**Fig. 1:**
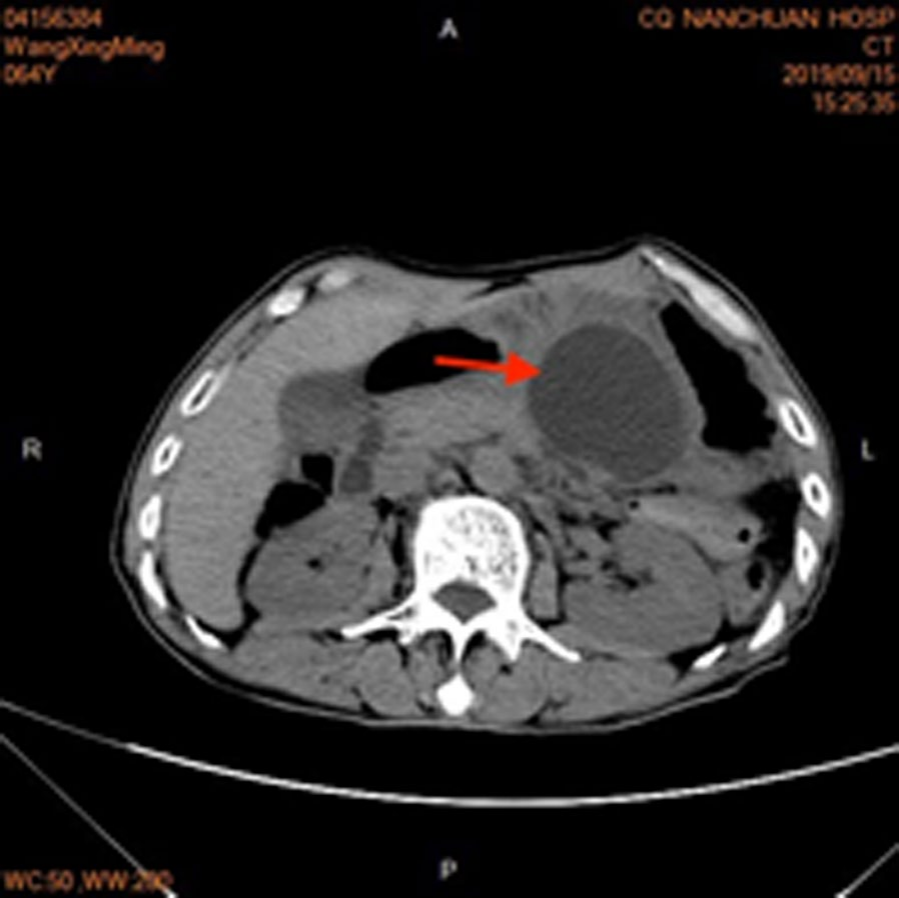
Abdominal CT revealed pancreatitis with a pseudocyst (red arrow) with a diameter measuring 7 cm

Contrast-enhanced abdominal computed tomography (CT) revealed pancreatitis with a pseudocyst (diameter 7 cm), in which there was fluid (Fig. 1). In the cyst, no necrotic tissue was found and the boundary was clear. Additionally, no obvious low-density mass was found around the cyst and the tail of pancreas was atrophied. The diameter of the pancreatic duct was approximately 0.45 cm. There were no enlarged lymph nodes in the upper abdomen. In addition, the CT scan of the chest was normal. MRCP revealed that there was no debris in the cyst, the pancreatic duct in the head of pancreas was not dilated, and the main pancreatic duct was not connected with the cyst.

**Fig. 2:**
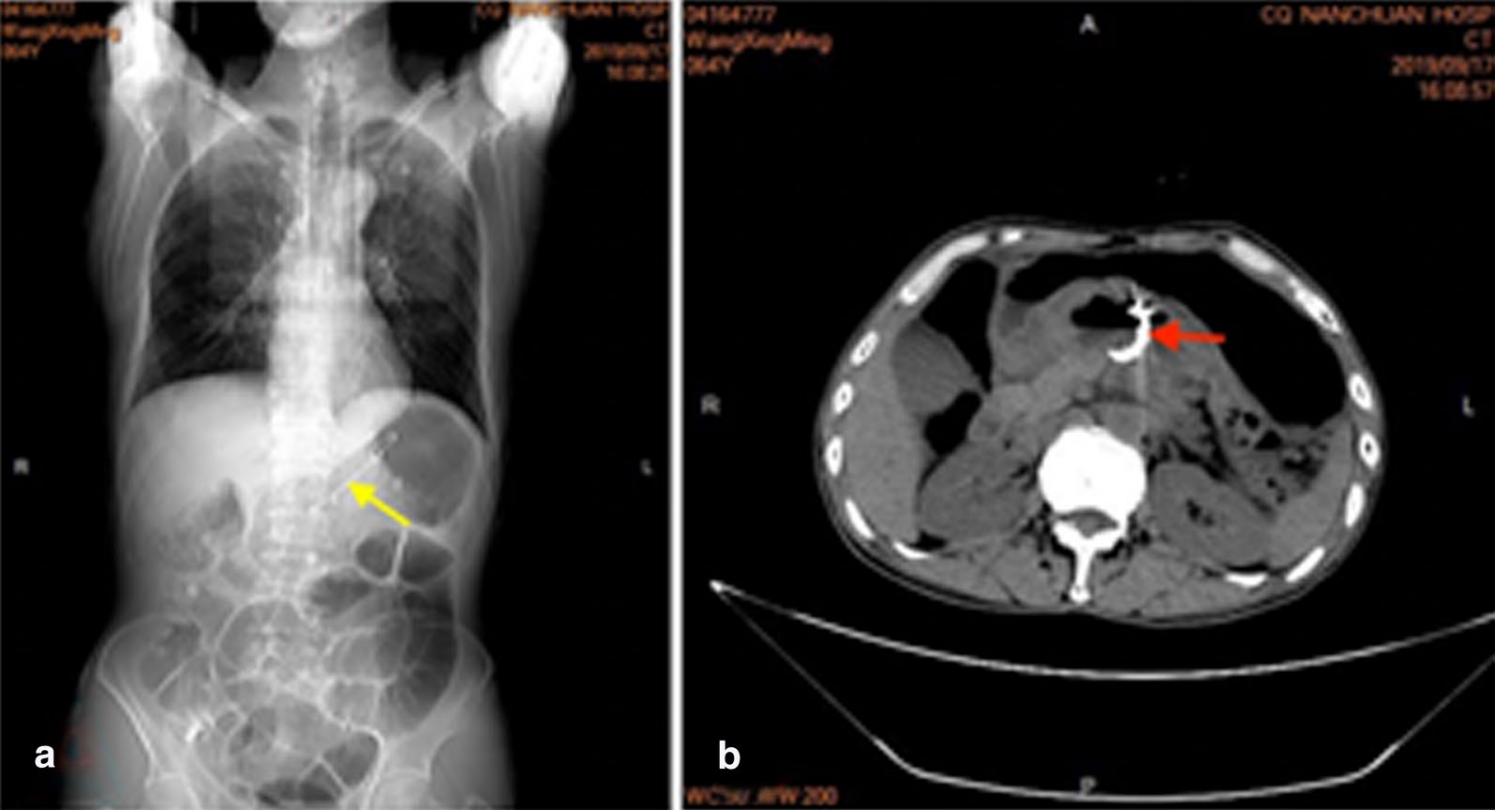
A plastic stent was placed for drainage of the pseudocyst through the pancreas and stomach. **a** The double pigtail plastic stent (yellow arrow) under X-ray. **b** One tail of the stent (red arrow) is located in the pseudocyst

Gastric endoscopy showed no abnormal findings in the upper gastrointestinal tract. During the linear EUS examination, a large pseudocyst was observed in the body of the pancreas with a maximum US cross-section of approximately 5.4 × 5.2 cm. The margin of the cyst was clear and no blood flow was detected with color Doppler ultrasonography. Some hyperechoic floccules were observed in the cyst and there was septum in the lumen. There was no debris in the cyst, and the cyst fluid aspirated by EUS-FNA was also without debris. There was no obvious dilatation of the pancreatic duct. Endoscopic ultrasound-guided fine-needle aspiration (EUS-FNA) was performed. The exact site of EUS-FNA was the posterior wall of the upper part of the stomach, and the distance between the ultrasonic probe and the cyst was no more than 1 cm. After puncturing the cystic lesion, the stylet was removed, and 10 mL of negative pressure suction was applied within the lesion. The syringe was filled with brown liquid. We collected 20 ml of fluid for the examination of amylase, carcinoembryonic antigen (CEA), and CA19-9. We also performed EUS-FNA to obtain specimens for histopathology and placed a plastic stent for drainage of the pseudocyst through the pancreas and stomach (Fig. 2). Puncture fluid examination revealed the following: CA19-9 > 12,000 U/ml, CEA 7097.42 ng/ml, amylase 27,145.3 U/L, and lipase > 6000 U/L.

**Fig. 3:**
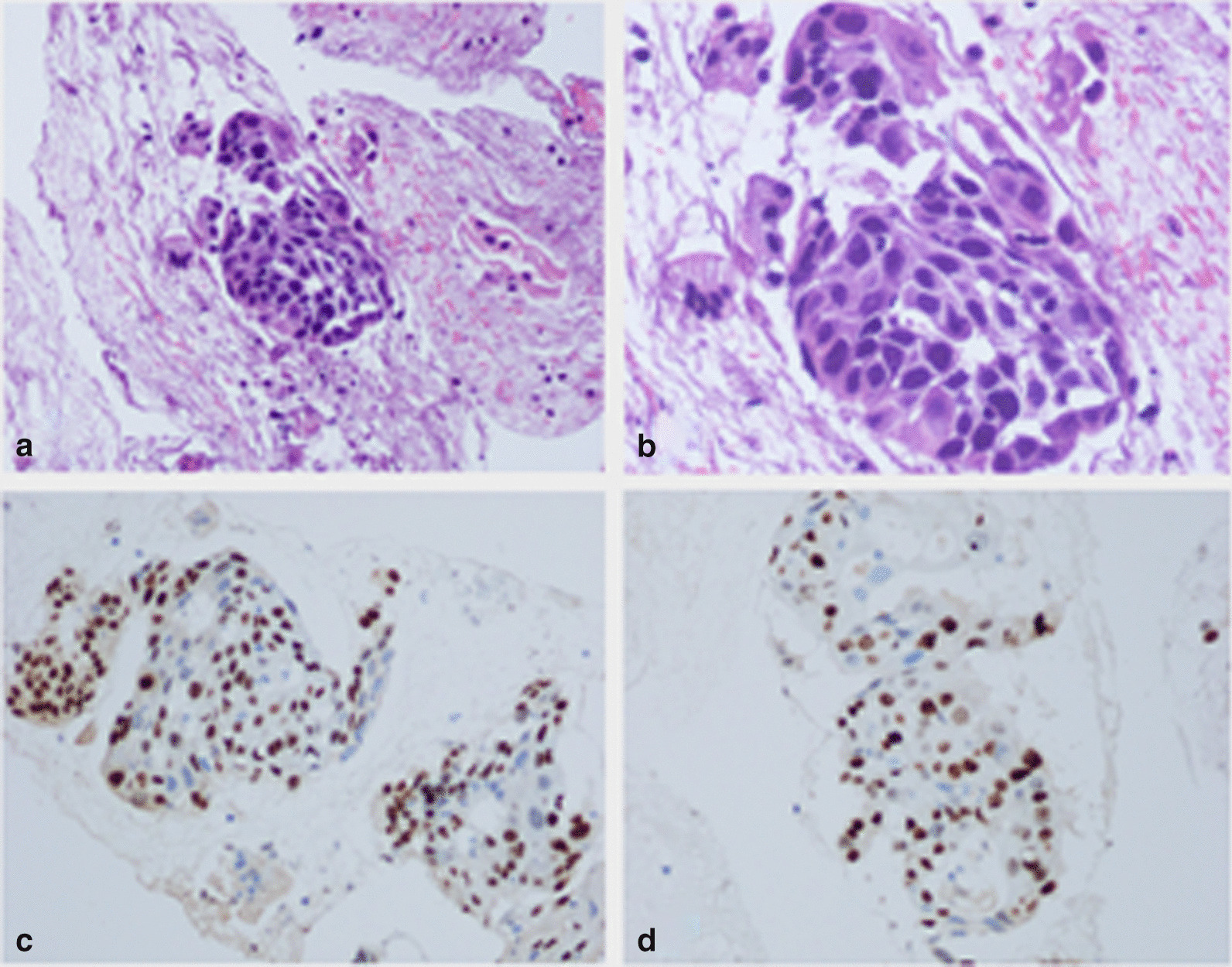
Based on histopathology combined with immunohistochemistry, the patient was diagnosed with squamous cell carcinoma (**a** HE × 100, **b** HE × 200). Immunohistochemical analysis showed the following: P40 ( +) (**c**) and Ki-67 (30%) (**d**)

After EUS-FNA, pathological cytology revealed an atypical cell mass, and cancer was suspected. Based on the immunohistochemistry results, CK ( +), P40 ( +), p63 ( +), CK7 (-) and Ki-67 (30%), the patient was diagnosed as squamous cell carcinoma (SCC) (Fig. 3).

The patient refused surgery, radiotherapy and chemotherapy. After drainage, the cyst shrank, but the patient died 3 months after diagnosis, due to liver metastasis and multiple organ failure.

## Summary of previously reported cases from 2009 to 2020

We searched PubMed for the following keywords in the title/abstract and found 15 cases of SCC of the pancreas reported from 2009 to 2020: “squamous cell carcinoma”, “pancreas”, “endoscopic ultrasound-guided fine-needle aspiration” and “EUS-FNA” [8–23]. A summary of these cases is displayed (Table 1). The patients ranged in age from 45 to 82 years, with a mean age of 67.9 ± 6.7 years. Among these 15 patients, only one had cystic lesions, which was diagnosed by EUS-guided confocal endoscopy, 1 had cystic and solid lesions, and the others had solid masses of the pancreas. There were no reports on pseudocyst-associated pancreatic SCC. Among the 15 patients, more than 50% (8/15) had metastasis at diagnosis. Although the patients received different treatments, including surgery, radiotherapy and chemotherapy, the survival time of almost all patients was less than 12 months. The prognosis of pancreatic SCC is poor.

**Table 1:** Clinical features of 15 cases with pancreatic squamous cell carcinoma diagnosed by EUS-FNA

Year	First author	Diagnostic mode		Metastasis	Therapy	Surviva*(months)
		CT	EUS and pathology			
2009[8]	Larry Hin Lai	Partial cystic mass in the tail of the pancreas	FNA/Cytopathology	Liver	-	-
2009[9]	Santiago SotoIglesias	Pancreatic mass	FNA/Cytopathology	None	Chemotherapy/radiation	12
2013[10]	Sepideh Nikfam	Solid mass of pancreatic body / tail	FNA/Cytopathology/Immunopathology	Liver and carina of the trachea	Chemotherapy	9
2014[11]	Alan Brijbassie	Cystic lesions of uncinate process of pancreas	FNA/Cytopathology	Adjacent lymphonodus	Radiation	-
2015[12]	Mudresh Mehta1	Pancreatic tail mass	FNA/Cytopathology/Immunopathology	None	-	2
2016[13]	Kyle Rowe	Pancreatic head mass, secondary cystic dilatation of pancreatic tail	FNA/Cytopathology	Liver	-	-
2017[14]	Rohan M.Modi	Cystic disease of pancreas	EUS-guided needle-based confocal laser endomicroscopy (nCLE) with an AQ-Flex 19 miniprobe/Cytopathology	Liver	-	-
2017[15]	Fernando Martínez de Juan	Solid and cystic mass of pancreas	FNA/Cytopathology and Partial pancreatectomy: Cytopathology/Immunopathology	Adjacent lymphonodusl	Surgery	> 5
2017[16]	Amir Kashani	Pancreatic tail mass	EUS-CNB/Cytopathology//Immunopathology	Liver	-	-
2017[17]	Bader A Alajlan	Pancreatic tail mass	FNA/Cytopathology	None	-	3
2017[18]	Ryan Glass	Cystic mass of head of pancreas	FNA/Cytopathology/Immunopathology	-	Chemotherapy	> 4
2017[19]	Seyed Hassan Abedi	Mass of body and tail of pancreas	EUS-FNA/Cytopathology/Immunopathology	-	-	8
2017[20]	Diogo Turiani Hourneaux De Moura	Solid mass of head of pancreas	EUS-FNA/Cytopathology	-	ERCP and entirely covered metallic stent	1
2018[21]	Ge Zhang	Pancreatic mass	EUS-FNA/Cytopathology	Gastric wall and Liver	Radiation	8
2019[22]	Nikolaos Machairas	Pancreatic head mass	EUS-FNA/Cytopathology	None	Surgery	2

## Discussion and conclusions

SCC is a very rare exocrine pancreatic malignant tumor. The clinical presentation of primary SCC of the pancreas is similar to that of ductal adenocarcinoma of the pancreas [24]. In 2019, Tella SH et al. searched the National Cancer Database (NCDB) (2004–15) and reported 182,090 patients prior to matching, 181,575 with pancreatic adenocarcinoma and 515 with SCC; thus, SCC accounted for only 0.28% of all pancreatic cancers. The proportions of patients with stage III and IV disease at diagnosis were 14% and 62%, respectively. For stage IV disease, the most common distant sites of metastases were the liver (32%) and lungs (6%) [25]. Although different therapeutic methods have been used to treat pancreatic SCC, including surgical resection, chemotherapeutic regimens, and radiotherapy, none have proven effective [26]. The proportion of patients with stage III and IV disease at diagnosis was above 76%, so survival was poor [25, 26].

EUS-FNA has been increasingly used for the identification of solid pancreatic lesions and has shown high sensitivity and specificity [5, 6]. EUS-FNA is also helpful for obtaining histopathology and cytology results to improve diagnostic accuracy in pancreatic cystic neoplasms. A combined analysis of cyst fluid CEA, CA19-9, and cyst fluid lipase levels provides the highest accuracy for differentiating malignant and benign pancreatic cystic lesionsn [27]. Pancreatic malignant cysts usually have the following characteristics: they are found in middle-aged and elderly people without a history of pancreatitis; imaging is often accompanied by separation, calcification, and pancreatic duct expansion; the contents of the cysts are mostly mucus, nipple, and solid components; and the level of amylase in the cysts is low [28]. However, in our case, there was no obvious sign of a malignant cyst at the initial examination. The patient experienced abdominal pain and was diagnosed with acute pancreatitis, which ultimately resulted in a large pseudocyst. During EUS-guided drainage, the color of the cyst fluid was brown, consistent with the appearance of a pseudocyst. Due to the increase in CA19-9, we performed EUS-FNA, but the final diagnosis was SCC of the pancreas. Disease progression was also consistent with that of pancreatic SCC.

Normally, the pancreas lacks squamous epithelium. Although there are many hypotheses, the pathogenesis of pure SCC of the pancreas remains elusive [24]. However, for patients with pancreatic pseudocysts with elevated serum CEA and CA19-9 levels, we should not rule out pancreatic cancer, which may also be a manifestation of primary pancreatic SCC. Additional molecular biological studies are needed to confirm these findings.

## Data Availability

This case report contains clinical data from the electronic medical record in The People’s Hospital of Nanchuan, Chongqing, China. Additional information is available from the corresponding author on reasonable request from the editor.

## Abbreviations

SCC: Squamous cell carcinoma
EUS-FNA: Endoscopic ultrasound-guided fine-needle aspiration
CA19-9: Carbohydrate antigen 19–9
CEA: Carcinoembryonic antigen
CT: Computed tomography
